# Mitomycin‐C treatment during differentiation of induced pluripotent stem cell‐derived dopamine neurons reduces proliferation without compromising survival or function in vivo

**DOI:** 10.1002/sctm.20-0014

**Published:** 2020-09-30

**Authors:** Benjamin M. Hiller, David J. Marmion, Rachel M. Gross, Cayla A. Thompson, Carrie A. Chavez, Patrik Brundin, Dustin R. Wakeman, Christopher W. McMahon, Jeffrey H. Kordower

**Affiliations:** ^1^ Department of Neurological Sciences Rush University Chicago Illinois USA; ^2^ College of Arts and Science, Vanderbilt University Nashville Tennessee USA; ^3^ Fujifilm Cellular Dynamics, Inc. Madison Wisconsin USA; ^4^ Center for Neurodegenerative Science, Van Andel Institute Grand Rapids Michigan USA; ^5^ Virscio, Inc. New Haven Connecticut USA; ^6^ Department of Psychiatry Yale School of Medicine New Haven Connecticut USA

**Keywords:** cell transplantation, immune‐deficient models, induced pluripotent stem cells, Parkinson's disease

## Abstract

Nongenetic methodologies to reduce undesirable proliferation would be valuable when generating dopamine neurons from stem cells for transplantation in Parkinson's disease (PD). To this end, we modified an established method for controlled differentiation of human induced pluripotent stem cells (iPSCs) into midbrain dopamine neurons using two distinct methods: omission of FGF8 or the in‐process use of the DNA cross‐linker mitomycin‐C (MMC). We transplanted the cells to athymic rats with unilateral 6‐hydroxydopamine lesions and monitored long‐term survival and function of the grafts. Transplants of cells manufactured using MMC had low proliferation while still permitting robust survival and function comparable to that seen with transplanted dopamine neurons derived using genetic drug selection. Conversely, cells manufactured without FGF8 survived transplantation but exhibited poor in vivo function. Our results suggest that MMC can be used to reduce the number of proliferative cells in stem cell‐derived postmitotic neuron preparations for use in PD cell therapy.

## Graphical abstract

In the present study, Kordower and colleagues offer evidence that, as an alternative to genetic selection methods, mitomycin‐C can be applied during the differentiation process of iPSC‐derived midbrain dopamine neurons as a means of removing proliferating cells without compromising graft health or function in the unilateral 6‐hydroxydopamine athymic rat model of Parkinson's disease.

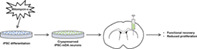

**Significance statement**

Nongenetic methodologies to reduce undesirable proliferation would be valuable when generating dopamine neurons from stem cells for transplantation in Parkinson's disease. To this end, this study modified an established method for differentiation of human induced pluripotent stem cells into midbrain dopamine neurons using an FDA‐approved chemotherapeutic (mitomycin‐C). These cells were transplanted to a rat model of Parkinson's disease and it is found that transplants of cells manufactured using mitomycin‐C had low proliferation while still permitting robust survival and function comparable dopamine neurons derived using genetic drug selection. The results of this study suggest that mitomycin‐C can be used to reduce the number of dividing cells in stem cell‐derived neuron preparations for use in Parkinson's disease cell therapy.

## 1 INTRODUCTION

Parkinson's disease (PD) is a progressive, debilitating neurodegenerative disease with over 60 000 new cases diagnosed each year in the United States. The current gold standard of care for PD, levodopa therapy, provides relief from motor symptoms for several years but ultimately results in motor fluctuations and dyskinesias. Therefore, it is critical to develop alternative therapeutic strategies that address the cardinal motor symptoms of PD in the long term. A vast body of preclinical studies, as well as small proof‐of‐principle clinical trials, have demonstrated that immature dopamine neurons grafted to the striatum can replace those lost in the substantia nigra and exert functional benefit.

In recent years, protocols have been developed to effectively direct differentiation of ESCs and induced pluripotent stem cells (iPSCs) toward a midbrain dopamine (mDA) neuron lineage.^1^, ^2^, ^3^, ^4^, ^5^ Importantly, when transplanted to rat and nonhuman primate PD models, dopamine neurons reinstate dopamine neurotransmission in the denervated striatum, restore motor function, and survive long‐term. We recently modified a so‐called “floorplate induction” differentiation protocol and demonstrated that human iPSCs differentiated to mDA neurons survive transplantation to the striatum of cyclosporine‐A‐immunosuppressed rats with unilateral 6‐hydroxydopamine (6‐OHDA) lesions, reinnervate the host, and elicit complete functional recovery of amphetamine‐induced motor asymmetry.^6^ The cryopreserved postmitotic iPSC‐mDA neurons we used contained a construct encoding neomycin resistance driven by the *Map2* promoter. Thus, we utilized Geneticin (G418) to select cells of neuronal lineage during the differentiation process. This protocol resulted in 97% of cells expressing the pan‐neuronal marker Map2 at 14 days post‐thaw in vitro. At 2 weeks after grafting, proliferation in transplants was negligible.^6^ However, due to potential safety concerns with clinical use of genetically engineered iPSC lines containing this type of drug selection cassette, this study evaluated nongenetic methodologies for iPSC‐mDA neuron purification to minimize proliferating cells. Various experimental cell therapies contain proliferative cells and while they are not necessarily dangerous, there have been reports of unexpected neural precursor outgrowth^4^, ^7^, ^8^ when engrafting cells made using previous protocols for differentiating dopamine neurons. Removal of proliferative cells from a postmitotic target cell population would address this risk. Antibody‐based methods have been described for enriching DA neuron populations, however, these methods target markers expressed on dopamine neurons or progenitors rather than specifically removing proliferative cells, and none of these markers are specific for the target population. For instance, sorting of CORIN‐expressing cells reduced the number of proliferative cells that emerged from a particular differentiation protocol^9^ but not all dopamine neuron progenitors express CORIN, and it has been reported that CORIN is not expressed on all target cells, in particular the midbrain DA progenitors with more caudal patterning,^10^ raising the possibility that CORIN sorting may remove desirable cells. Other surface markers that have been used for antibody‐based enrichment include NCAM, LRTM1, CD166, and IAP,^11^, ^12^, ^13^, ^14^ but none of these markers are specific to the target population. In addition, sorting with antibodies and magnetic beads greatly reduces the yield of the differentiation process, impeding scale‐up.

Mitotic inhibitors, DNA synthesis inhibitors, DNA cross‐linkers, and other selective agents have long been used to prevent aberrant outgrowth in culture. Agents such as cytosine arabinoside (Ara‐C) are commonly used to slow glial cell division in primary neuronal cultures.^15^, ^16^, ^17^ Therefore, we hypothesized that a similar approach could be used in‐process and at large scale during iPSC‐mDA neuron differentiation, providing that the selective agent was added when, or after, mDA neurons were leaving the cell cycle. After screening several such compounds, a low and sustained concentration of mitomycin‐C (MMC) was selected for in vivo testing. In addition, as an alternative approach we explored whether modifying the culture protocol by omitting FGF8 could result in a high yield of transplantable dopamine neurons without G418 selection. We found that both the addition of MMC and the omission of FGF8 generated large numbers of iPSC‐mDA neurons that could be cryopreserved. These cells were then transplanted to the striatum of athymic rats with unilateral 6‐OHDA‐lesions and monitored motor asymmetry, survival of grafted dopamine neurons, and cell proliferation in transplants up to 9 months. Omission of FGF8 during the cell differentiation protocol resulted in smaller and less functionally competent transplants with fewer surviving dopamine neurons. By contrast, treatment with an optimized concentration of MMC resulted in a significantly reduced number of proliferating cells in the transplants without compromising graft function or survival.

## 2 EXPERIMENTAL PROCEDURES

All animal procedures were performed with Institutional Animal Care and Use Committee approval from Rush University Medical Center.

All statistical analyses were performed using Prism (GraphPad). Data from immunohistochemical analyses were analyzed using a one‐way analysis of variance with Tukey's test post hoc. Behavioral data were analyzed with a two‐way analysis of variance and Tukey's test post hoc. Cell health data were analyzed using a Kruskal‐Wallis test followed by Dunn's post hoc. All animal data are expressed as mean ± SEM.

### 2.1 Cell differentiation, application of chemotherapeutic

Research use G418 neurons (iCell DopaNeurons, Fujifilm Cellular Dynamics, Inc.) were derived as previously described,^6^ using methods adapted from published reports.^3^, ^4^ Briefly, small molecule inhibitors of GSK‐3 (CHIR99021, Stemgent) and SMAD signaling (LDN‐193189, Stemgent, and SB431542, Sigma) and activation of the Hedgehog signaling pathway were used to pattern midbrain lineage dopamine neuron progenitors. Relevant to this report, the G418 differentiation protocol includes the use of FGF8b (100 ng/mL, Proteintech) during process days 11 to 17. In addition, the iPSC line was engineered with a neomycin resistance selection cassette driven by the human *Map2* promoter, and G418 was used in‐process to remove non‐neuronal cells. Progenitor cells were plated on process day 17 in maturation medium^4^ consisting of Neurobasal medium (Thermo), B27 minus Vitamin A (2%, Thermo), GlutaMAX (2 mM, Thermo), ascorbic acid (200 μM, Sigma), dibutyryl cAMP (500 μM, Sigma), brain‐derived neurotrophic factor (BDNF) (20 ng/mL, biotechne), glial cell line‐derived neurotrophic factor (GDNF) (20 ng/mL, biotechne), TNF‐β3 (1 ng/mL, biotechne), and N‐[N‐(3,5‐difluorophenacetyl)‐l‐alanyl]‐S‐phenylglycine t‐butyl ester (DAPT) (5 μM, Sigma). The cells were replated on process days 24 and 31, and postmitotic neurons were cryopreserved on process day 38. The protocol variations described in this report were made using the same iPSC line and with the same manufacturing procedure, but with the following modifications:

*Early Cryo*: Identical process to G418, except that the replate on process day 31 was omitted, and cryopreservation was performed on day 33.

*C50 and C125*: Identical process to “Early Cryo” cells, except that no G418 drug selection was used. Instead, MMC (Sigma) was added to maturation medium (50 or 125 ng/mL final concentration) immediately before feeding cells on process days 27 and 29. Cryopreserved on process day 33.

*NoFGF8*: This process modification omitted the use of FGF8b, G418, or MMC drug selection. Progenitors were plated in maturation medium earlier (process day 13) because the FGF8‐driven progenitor expansion phase was removed. Cells were replated on process day 19, and cryopreserved on day 26.

### 2.2 Lesion and transplant

Female athymic rats (9‐10 weeks old, 170‐200 g) received unilateral injections of 6‐OHDA (15 mg in 3 μL 0.5% ascorbic acid) to the right MFB (Anterior/Posterior [AP]: −4.0 mm; Medial/Lateral [ML]: −1.3 mm from bregma, Dorsal/Ventral [DV]: −7.0 mm from dura). Animals with confirmed lesions 10 weeks following 6‐OHDA injection received striatal (AP: +0.5 mm; ML: ±3.0 mm from bregma, DV: −5.3 mm from dura) injections of iPSC‐mDA neurons (N = 8‐11/group) and were sacrificed at 9 months post‐transplantation. In short‐term survival studies, female (3 months, 6‐OHDA‐lesioned/behaviorally asymptomatic) or male (2 weeks, intact) Sprague Dawley rats were immunosuppressed with cyclosporine‐A (10 mg/kg, i.p., q24h) beginning 2 days pretransplantation (AP: +0.6 mm; ML: ±3.0 mm from bregma, DV: −5.3 mm from dura). All grafts were 3 μL (1.5 × 10^5^ cells/μL).

## 3 RESULTS

### 3.1 Alternative purification methods for iPSC‐mDA neurons

In order to identify a suitable agent that would allow us to select for postmitotic cells only, we tested the efficacy of several compounds that have been shown to selectively inhibit the growth of dividing cells, including amiodarone,^18^ cytosine arabinoside (Ara‐C),^15^ and MMC.^19^ Several of these compounds have been approved by the FDA for chemotherapeutic use, such as treatment of pancreatic and gastric adenocarcinoma with MMC, or treatment of leukemias and lymphomas with Ara‐C. For each compound, we tested different concentrations applied for different periods of time, focusing on the window when neuron progenitors begin to leave the cell cycle (as measured by onset of MAP2 expression, process day 26), and before cryopreservation (process day 33‐38). We added the compounds to the medium immediately before every‐other‐day feeds, with total time of exposure between 2 and 5 days. For MMC, we also tested high concentrations administered for a short period of time (5‐10 μg/mL for 1‐2 hours), akin to how feeder cells are mitotically inactivated.^20^ After multiple experiments, we rejected amiodarone and Ara‐C because the conditions tested resulted in low process yields compared to controls (G418 drug selection), and/or were unable to remove proliferative cells, as evidenced by a decrease in neuron purity as the cells were cultured post‐thaw (Table S1). However, conditions were identified using the DNA cross‐linker MMC that resulted in a relatively pure postmitotic neuron population by the end of the differentiation process without negatively affecting yield or apparent health of the neurons. Experiments done at small scale identified the optimal treatment window (process days 27‐31) and concentration range (≤500 ng/mL) for MMC treatment, and follow‐up experiments narrowed the optimal concentration to 50 to 200 ng/mL, a range low enough to avoid toxicity to the postmitotic neurons when the process is carried out at larger scale (150‐1275 cm^2^). Under these conditions, neuron purity was maintained after post‐thaw culturing, with no visible outgrowth of proliferative cells (Table S1). Therefore, we chose this method for further testing.

We used the MMC method for purification to produce batches of cells with the same methods and at the same vessel scale used in the established, scaled‐up process (G418), substituting 50 ng/mL (C50) or 125 ng/mL (C125) MMC in place of G418 selection to remove proliferative cells. Based on our previous work^6^ and literature indicating that less mature dopamine neurons engraft more efficiently,^21^, ^22^ we cryopreserved these cells on process day 33, 5 days earlier than G418 cells. For direct comparison, we also manufactured a different batch of iPSC‐mDA neurons using the established process, including G418 treatment, but cryopreserved on process day 33 (Early Cryo). Protocol variations are detailed and diagrammed in Table S2 and Figure S1.

In addition, we adopted a differentiation protocol that does not require FGF8.^3^, ^4^ In the absence of FGF8, it was possible to omit the G418 selection step and still reach a high level of post‐thaw neuron purity with very few dividing cells. This protocol (NoFGF8), however, resulted in lower yields and cell viability, and a less desirable mDA neuron patterning profile (in particular, lower Engrailed 1 expression).

Therefore, cells were manufactured using this modified process as an alternative method of generating cells without need for a purification step. Because it lacked an FGF8 expansion phase, we shortened the overall progenitor phase, and cryopreserved on process day 26 when the cells had reached approximately the same maturation stage as cells made utilizing FGF8 and cryopreserved on day 33. Each cell type (NoFGF8, C50, C125, Early Cryo, and G418) was evaluated in vitro and in vivo.

### 3.2 In vitro analysis

**FIGURE 1:**
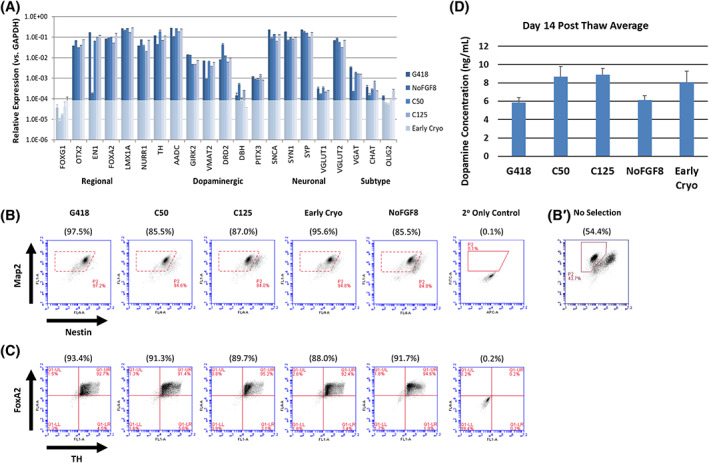
In vitro cell characterization. A, quantitative polymerase chain reaction comparing mRNA expression of regional, dopaminergic neuron, pan‐neuronal, and cell subtype markers in iPSC‐mDA neurons made using different manufacturing procedures. mRNA was isolated from cells immediately post‐thaw. Values are expressed as relative to glyceraldehyde‐3‐phosphate dehydrogenase (GAPDH), from triplicate technical replicates (mean ± SEM). The results are representative of two experiments, and gene expression values from G418 cells are similar to those previously reported.^6^ Values <10^−4^ were considered background. B, Intracellular staining and flow cytometry of iPSC‐mDA neurons at 3 days post‐thaw. Map2 (*y*‐axis) vs Nestin (*x*‐axis). Two independent thaws were performed for each cell type, with average values of the Map2+/Nestin‐ and FoxA2+/TH+ populations shown in parentheses. For comparison, Map2/Nestin staining of G418 cells differentiated without the G418 selection step (no selection) are shown (B′, separate experiment). C, FoxA2 (*y*‐axis) vs Tyrosine Hydroxylase (*x*‐axis). Staining of cells with isotype control were used to set FoxA2/TH quadrants. D, Dopamine secretion by iPSC‐mDA neurons. ELISA for dopamine following 30 minutes KCl depolarization at 14 days post‐thaw. Data graphed as mean ± SEM (N = 3 biological samples with duplicate technical replicates assayed per condition). iPSCs, induced pluripotent stem cells; mDA, midbrain dopamine

We previously demonstrated that human iPSC‐mDA neurons (G418) possess a gene expression profile similar to human substantia nigra, with high expression of markers for midbrain floor‐plate regionalization (FOXA2, LMX1A, EN1, OTX2) and dopamine neurons (TH, NURR1, GIRK2, PITX3, DRD2, VMAT2, AADC), and low expression of markers for forebrain (FOXG1) or other cell subtypes (DBH, vGLUT1, CHAT, OLIG2).^6^ In the current study, we used the same quantitative polymerase chain reaction panel (Table S3) to compare the expression of markers in iPSC‐mDA neurons produced using the different manufacturing procedures (Figure 1A). The results show that the addition of MMC as a purification agent did not have a major effect on the expression of any monitored genes. However, excluding FGF8 from the differentiation process resulted in significantly reduced expression of transcription factor Engrailed‐1 (EN1), which plays an important role in the development and maintenance of mDA neurons,^23^ and a less marked reduction in expression of VMAT2 and VGAT.

Using intracellular staining and flow cytometry, we assessed the purity and maturity of iPSC‐mDA neurons we obtained with different culture protocols at 3 days post‐thaw (Figure 1B). All manufacturing methods resulted in a majority (84%‐97%) of cells exhibiting characteristics of postmitotic neurons (Map2+/Nestin−). The NoFGF8 and C125 preparations contained more than 10% Nestin+ cells, but we found that these cells were not rapidly dividing and neuron purity (%Map2^+^/Nestin^−^) was maintained after extended culturing (Table S1). In contrast, cells manufactured using the established G418 process, but without G418 selection against cells not expressing Map2 (No Selection, B′), contained a large population (54.4%) of Nestin+ cells, many of which continued to divide after in vitro culturing. We did not fully characterize this cell population, but found that a significant portion express markers of glial and astrocyte precursors including CD44 and GLAST (data not shown).

In all cell manufacturing protocols, over 90% of cryopreserved cells expressed FoxA2, indicating they were of floor plate identity. Similarly, around 93% of cells were immunoreactive for tyrosine hydroxylase (TH‐ir) irrespective of protocol, suggesting that the different methods did not significantly impact dopamine neuron purity and survival up to and beyond the point of cryopreservation (Figure 1C). However, it should be noted that the culture conditions induce TH expression in nondopamine neurons,^24^ so whether dopamine neurons are specifically affected cannot be stated conclusively.

In the next set of experiments, we evaluated cells after 2 weeks of in vitro maturation post‐thaw. We monitored iPSC‐mDA neuron function by measuring dopamine secretion in culture medium (Figure 1D). Consistent with our earlier work,^6^ dopamine secretion by G418 cells was detected after depolarization using high potassium. Comparable levels of dopamine were secreted by all cell preparations, indicating that different manufacturing and purification strategies did not impact the ability of the cells to produce dopamine at 2 weeks post‐thaw.

High cell viability in vitro is required for cells to survive transplantation. Therefore, we used several post‐thaw cell health assessments to assess viability on iPSC‐mDA neurons produced using different manufacturing protocols (Figure S2). Cell recovery, after cryopreservation and thawing, exceeded 60% for all preparations. Furthermore, using a trypan blue‐exclusion test, the post‐thaw viability of all preparations was 75% to 93% (*P* < .05, NoFGF8 compared to C125). Plating efficiency measures how efficiently cells adhere to an extracellular matrix‐coated surface and survive over time, and is a more stringent measure of post‐thaw cell health compared to viability measured using trypan blue‐exclusion. The plating efficiencies of different iPSC‐mDA neuron preparations were 58% to 69%. The viability of plated cells after 3 days in vitro (DIV) was 86% to 94% as assessed by trypan blue (*P* < .05, G418 compared to Early Cryo). Taken together, the measurements suggest that the post‐thaw cell health of the cells made using different manufacturing methods is similar to the well‐characterized G418 cells.

### 3.3 In vivo efficacy

After in vitro analyses indicated that MMC‐treated cells resulted in mDA neurons with high viability, we tested their ability to survive intracerebral transplantation. In a first experiment, we transplanted neurons derived from MMC‐treated (C50 and C125) iPSCs bilaterally to the striatum of immunosuppressed Sprague‐Dawley rats without 6‐OHDA lesions. Three months post‐transplantation, we identified surviving grafts in brain sections from all animals using antibodies specific to human nuclei (HuNuclei) and neural cell adhesion molecule (HuNCAM; Figure S3). High magnification images revealed that both cell types/groups project HuNCAM‐ir fibers from the graft into the host striatum (Figures S3A′ and S3B′). Furthermore, all grafts contained TH‐ir neurons, indicating a dopaminergic phenotype (Figures S3A″ and S3B″).

After determining that MMC‐treated iPSCs survive short‐term in intact immunosuppressed rats, we next tested the efficacy of each cell type in athymic rats with a unilateral 6‐OHDA lesion of the nigrostriatal pathway. Briefly, we made intrastriatal injections of G418, C50, C125, Early Cryo, or NoFGF8 cells ipsilateral to the lesion. We tested d‐amphetamine‐induced rotations every 2 months, sacrificed rats 9 months post‐transplantation, and performed immunohistochemistry on free‐floating coronal sections to evaluate graft survival and innervation.

**FIGURE 2:**
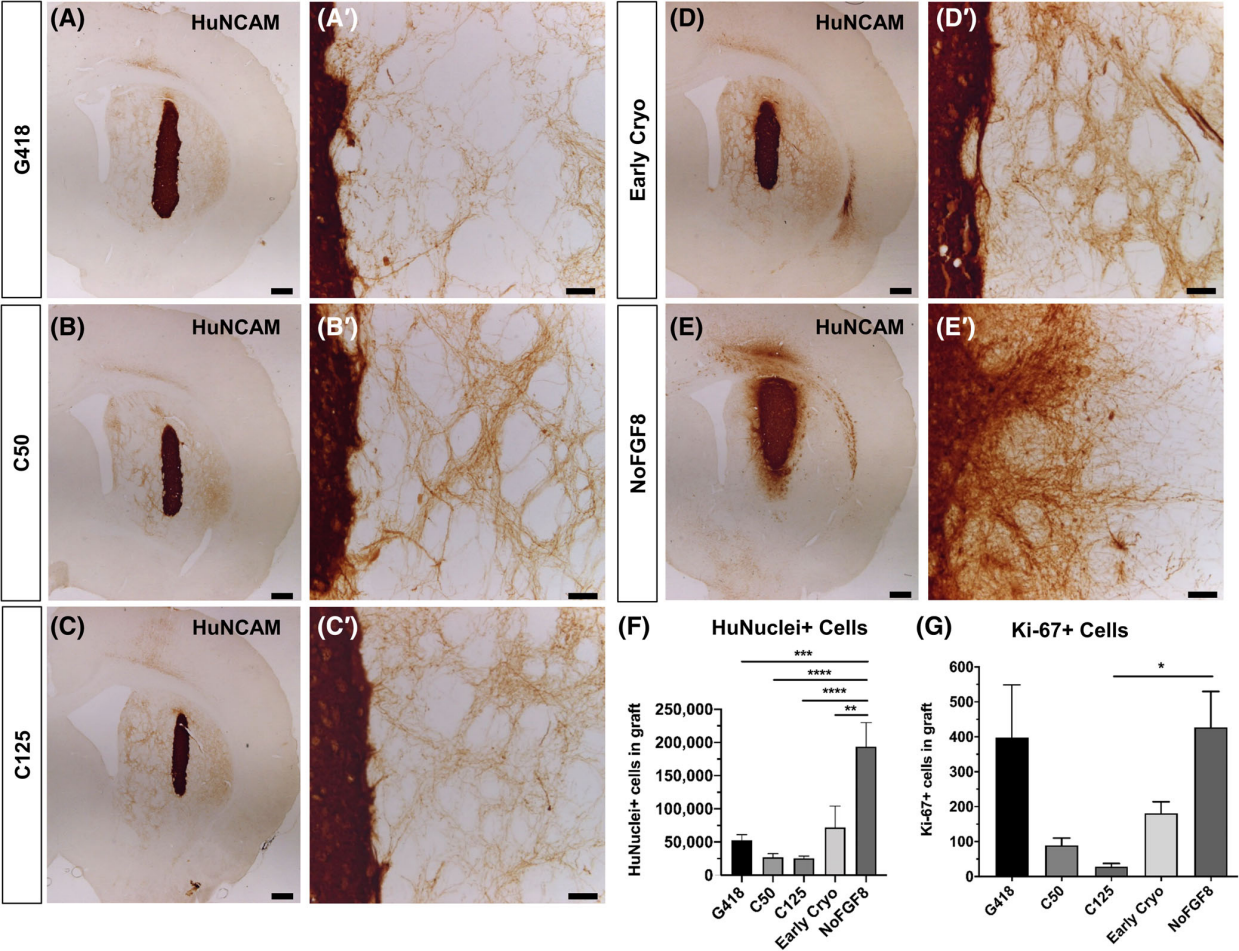
Graft analysis 9 months post‐transplantation. A‐E, 6‐OHDA lesioned athymic rats transplanted with G418 (A, A′), C50 (B, B′), C125 (C, C′), Early Cryo (D, D′), or NoFGF8 (E, E′) iPSC‐derived dopaminergic neurons and sacrificed 9 months post‐transplantation. Fiber outgrowth was markedly increased relative to 3 month animals (Figure S3) and covered a large portion of the striatum with the exception of NoFGF8. F,G, Stereological quantification of HuNuclei (F) and Ki‐67 (G). The number of HuNuclei‐ir (F) and Ki‐67‐ir (G) cells was quantified by stereological analysis via StereoInvestigator. The group with the most surviving HuNuclei‐ir cells was NoFGF followed by Early Cryo (*P* < .005 compared to NoFGF8), G418 (*P* < .001 compared to NoFGF8), C50 (*P* < .0001 compared to NoFGF8), and C125 (*P* < .0001 compared to NoFGF8). Cells treated with the MMC (C50 and C125) had the fewest numbers of Ki67‐ir cells (*P* = .051 and *P* < .05 compared to NoFGF8, respectively). Scale bar = 500 μm (A, B, C, D, E) and 50 μm (A′, B′, C′, D′, E′). 6‐OHDA, 6‐hydroxydopamine; iPSC, induced pluripotent stem cell; MMC, mitomycin‐C

HuNCAM immunostaining revealed surviving grafts (Figure 2A‐E) in all animals. Transplanted cells in all groups except the NoFGF8 group, exhibited dense, extensive HuNCAM‐ir fibers projecting from the graft into the host striatum, including the dorsolateral region. The NoFGF8 grafts appeared larger than those from all other groups and displayed dense HuNCAM‐ir fibers exiting the graft body; however, these fibers only extended approximately 200 μm from the edge of the graft into the host parenchyma (Figure 2E′), leaving much of the striatum devoid of graft‐derived innervation.

We immunohistochemically stained graft sections for HuNuclei and utilized unbiased stereology to estimate the total number of surviving cells. We found a mean of 210 929 ± 36 198 NoFGF8 cells, 70 511 ± 28 861 Early Cryo cells (*P* < .01 compared to NoFGF8), 60 266 ± 11 093 surviving G418 (*P* < .001 compared to NoFGF8), 30 479 ± 4333 C50 cells (*P* < .0001 compared to NoFGF8), and 25 605 ± 3168 C125 cells (*P* < .0001 compared to NoFGF8) (Figure 2F). Importantly, all HuNuclei‐ir cells were located within the graft tissue and none were seen dispersed from the graft core.

In order to assess the risk for tumorigenesis, we immunostained sections for Ki67, a marker for dividing cells. In line with previous studies,^6^ we found few proliferating K67‐ir cells (Figure 2G) in any grafts. Cells treated with MMC (C50 and C125) had the fewest numbers of Ki67‐ir cells (179 ± 45, *P* = .051 compared to NoFGF8 and 66 ± 26, *P* < .05 compared to NoFGF8, respectively) compared to G418 (1020 ± 368), Early Cryo (421 ± 95), and NoFGF8 (1740 ± 456). We never observed dividing cells clustered together, indicating that cells were not undergoing multiple rounds of division.

**FIGURE 3:**
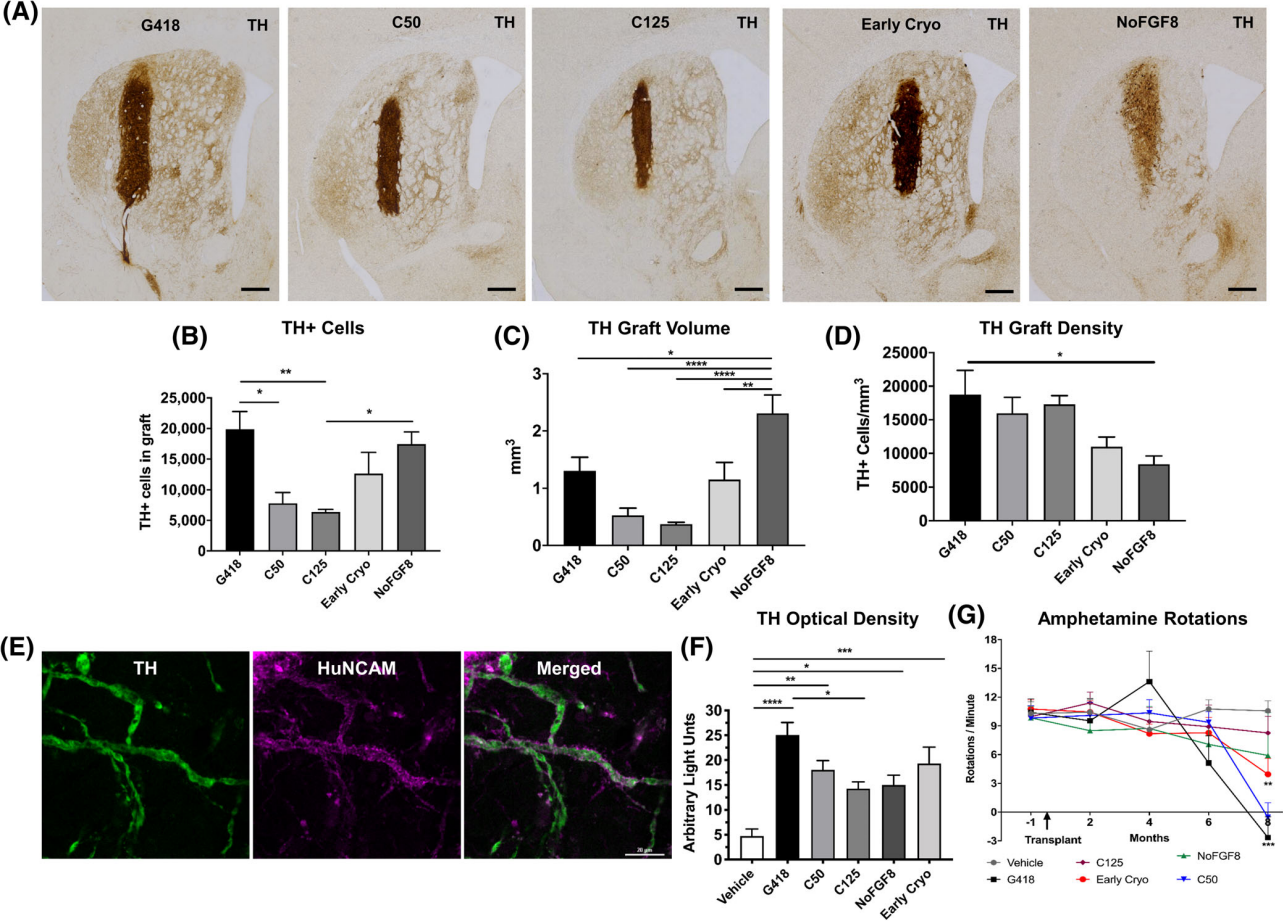
Cell survival and rescue of motor deficits in vivo. A, 6‐OHDA‐lesioned athymic rats 9 months post‐transplantation stained for TH. Grafts were seen in the dorsolateral striatum with fibers extending from the graft core into the host striatum with the exception of NoFGF8. B, Unbiased stereology was used to quantify TH‐ir cells in grafted animals, and showed that C50 (*P* < .05 compared to Early Cryo) and C125 (*P* < .05 compared to NoFGF8, *P* < .005 compared to Early Cryo) had the lowest numbers of TH‐ir cells in each graft. C, Analysis of graft volume in mm^3^. NoFGF8 grafts had larger volumes compared to other cell types (*P* < .05 compared to G418, *P* < .1 compared to Early Cryo, *P* < .001 compared to C50 and C125). D, Density of TH‐ir cells was calculated using stereological estimates and graft volume. NoFGF8 grafts had the lowest density of TH‐ir cells/mm^3^ (*P* < .05 compared to G418). E, Confocal imaging of TH/HuNCAM double‐labeled fibers in the striatum. Complete colocalization indicates that TH fibers in the striatum are of human origin and thus graft‐derived. F, Optical densitometric analysis of TH‐ir fibers was performed and staining intensity was measured in arbitrary units (AU). G418 (*P* < .0001), Early Cryo (*P* < .001), C50 (*P* < .005), NoFGF8 and C125 (*P* < .05) groups showed significant differences in TH‐ir fibers measured in the striatum compared to vehicle. G, Motor asymmetry was analyzed preoperatively and every 2 months following transplantation. Rats that received G418 (*P* < .001 compared to vehicle) or C50 (*P* < .005 compared to vehicle) grafts fully recovered motor asymmetry by 8 months post‐transplantation. Scale bars = 500 μm (A) and 20 μm (E). 6‐OHDA, 6‐hydroxydopamine

We then quantified the number of surviving TH‐ir neurons in the grafts (Figure 3A,B). G418 grafts contained 19 861 ± 2902 TH‐ir cells. Grafts of C50 (7774 ± 1801, *P* < .05 compared to Early Cryo) and C125 (6536 ± 453, *P* < .05 compared to NoFGF8, *P* < .005 compared to Early Cryo) neurons yielded the lowest number of surviving TH‐ir neurons in the transplants. Interestingly, FGF8 patterning was not required to produce relatively large numbers of TH‐ir neurons in the graft (17 430 ± 2002) while Early Cryo grafts yielded 12 612 ± 3469 surviving TH‐ir neurons. Table 1 shows graft composition in terms of HuNuclei, TH, Ki‐67, and 5‐HT.

**TABLE 1:** Summary data

Mean number of:	Cell type				
	G418	C50	C125	Early Cryo	NoFGF8
Total surviving cells (HuNuclei), % of total injected^a^	60 266 13.39%	30 479 6.77%	25 605 5.69%	70 511 15.67%	210 929 46.87%
Dopamine neurons (TH), % of total surviving	19 861 37.16%	7774 25.40%	6356 27.98%	17 430 24.22	17 430 12.99%
Proliferating cells (Ki‐67), % of total injected	1020 0.23%	178.7 0.04%	66 0.01%	421.2 0.09%	1740 0.39%
Serotonergic cells (5‐HT)	230	36	46	106	147
Amphetamine‐induced rotations/minute, % reduction from vehicle^b^	125.09%	105.14%	21.84%	62.65%	44.01%

^a^ 450 000 cells were transplanted.

^b^ At 8 months post‐transplantation; values over 100% indicate negative overshoot; vehicle rotations/minute at 8 months post‐transplantation was 10.57 ± 1.05; baseline rotations/minute for all groups was 10.22 ± 0.18 (mean ± SEM).

We assessed graft volume in TH‐immunostained sections and found that MMC‐treated grafts were smallest (*P* < .0001; C50 and C125 compared to NoFGF8), followed by Early Cryo (*P* < .01 compared to NoFGF8), G418 (*P* < .05 compared to NoFGF8), and NoFGF8 (Figure 3C). Despite this, NoFGF8 grafts had fewer TH‐ir neurons/mm^3^ compared to G418 (*P* < .05, Figure 3D).

We next evaluated the density of graft‐derived TH‐ir fiber innervation of the host striatum, because mere survival of TH neurons is insufficient for grafts to exert functional effects on motor deficits.^25^ In TH‐immunostained sections, we measured optical density (OD) throughout the rostrocaudal extent of the striatum, omitting grafted cells. All groups except C125‐treated animals showed significant increases in TH OD measurements compared to vehicle control (C50 *P* < .01, Early Cryo *P* < .001, NoFGF8 *P* < .05; Figure 3F). The graft‐derived reinnervation was significantly greater for the G418 group (*P* < .05, compared to C125). To validate that the TH fibers we measured were in fact graft‐derived and not fibers persisting after 6‐OHDA, we performed confocal microscopy on TH/HuNCAM double labeled sections. We found that TH‐ and HuNCAM‐ir fibers colocalized, confirming that these fibers were of human origin and graft‐derived (Figure 3E). Based on the robust survival of NoFGF8 grafts, it was surprising that so few TH‐ir fibers emanated from the grafts. This observation suggests that while overall cell survival did increase in NoFGF8 grafts, graft‐derived TH fiber innervation of the host striatum declined in the absence of FGF8 patterning. Additionally, data on TH fiber outgrowth indicate that treatment with 50 ng/mL MMC did not reduce the capability of engrafted cells to project dopaminergic fibers into the host striatum.

To evaluate recovery of lesion‐induced motor asymmetry, we performed amphetamine‐induced rotation testing every 2 months (Figure 3G). Animals receiving either G418 neurons or C50 grafts displayed complete reversal in amphetamine‐induced rotations at 8 months post‐transplantation compared to vehicle (*P* < .01, *P* < .001, respectively; Figure 3E). Additionally, the Early Cryo group achieved partial reversal (*P* < .05) at 8 months post‐transplantation. Interestingly, the C125 group showed little change in rotations at 8 months post‐transplant. The data suggest that exposing the iPSCs to a low (C50), but not a high (C125), concentration of MMC does not alter the capacity of the iPSC‐mDA neurons to survive and exert functional effects in behavioral tests after grafting to rats with a unilateral 6‐OHDA lesion.

In a separate cohort of cyclosporine‐A‐immunosuppressed Sprague Dawley rats, we tested whether culturing for 2 DIV affected the outcome of the grafts in a short‐term survival study. At 2 weeks post‐transplantation, we found no significant differences in the number of surviving TH‐ir neurons between G418 neurons cultured 2 DIV and those engrafted directly from the cryovial (Figure S4). However, we did observe a marked reduction in the number of surviving TH‐ir cells in NoFGF8 grafts (*P* < .05), demonstrating that those cells are more suitable for transplantation when grafted immediately after thawing compared to after culturing them for 2 days after thawing.

### 3.4 Graft characterization

**FIGURE 4:**
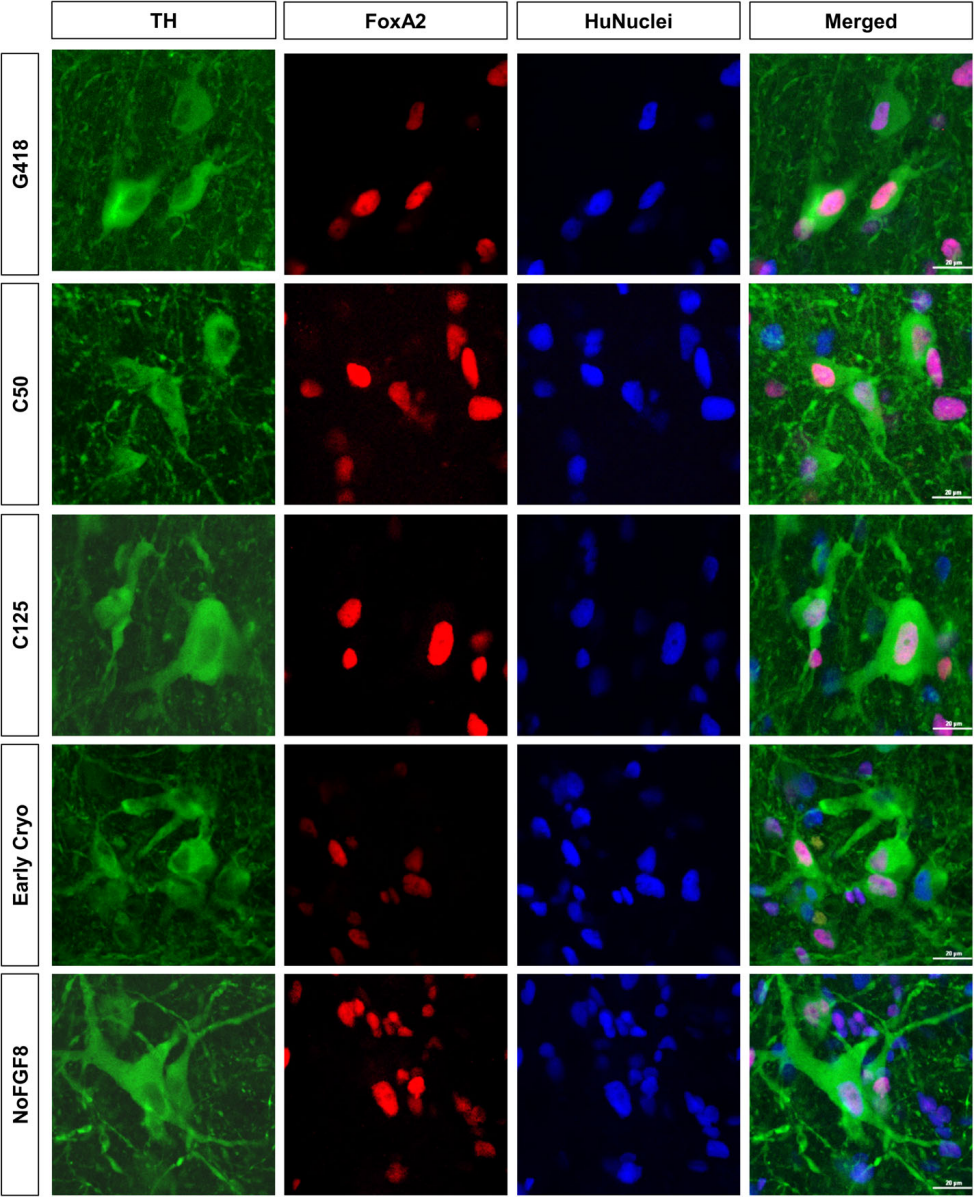
Maintenance of midbrain dopaminergic lineage. Confocal images of representative sections triple labeled for TH (green), FoxA2 (red), and HuNuclei (blue). Confocal microscopic analysis revealed that the majority of TH‐ir neurons also expressed FoxA2, indicating midbrain floorplate origin and that the addition of MMC did not alter the phenotype of the transplanted neurons. Scale bar = 20 μm. MMC, mitomycin‐C

The transcription factor Forkhead Box A2 (FoxA2) has been shown to be a key factor in in inducing the birth of mDA neurons, as well as adult mDA neuron maintenance.^26^, ^27^ Given its central role in development and function of authentic mDA neurons,^4^ we utilized confocal microscopy to evaluate the FoxA2 expression in TH‐ir neurons. Across all groups, we observed a large majority of TH‐ir neurons coexpressing FoxA2 and HuNuclei (Figure 4), indicating that they were of human origin and maintain floorplate midbrain lineage of authentic mDA neurons. Of note, we also found some TH‐negative FoxA2‐ir HuNuclei‐ir neurons, which is in agreement with reports that our differentiation protocol, similar to those of others in the field, produces a heterogeneous mixture of closely‐related midbrain‐phenotype progenitor cells, of which a subset is destined to mature into mDA neurons.^28^, ^29^

**FIGURE 5:**
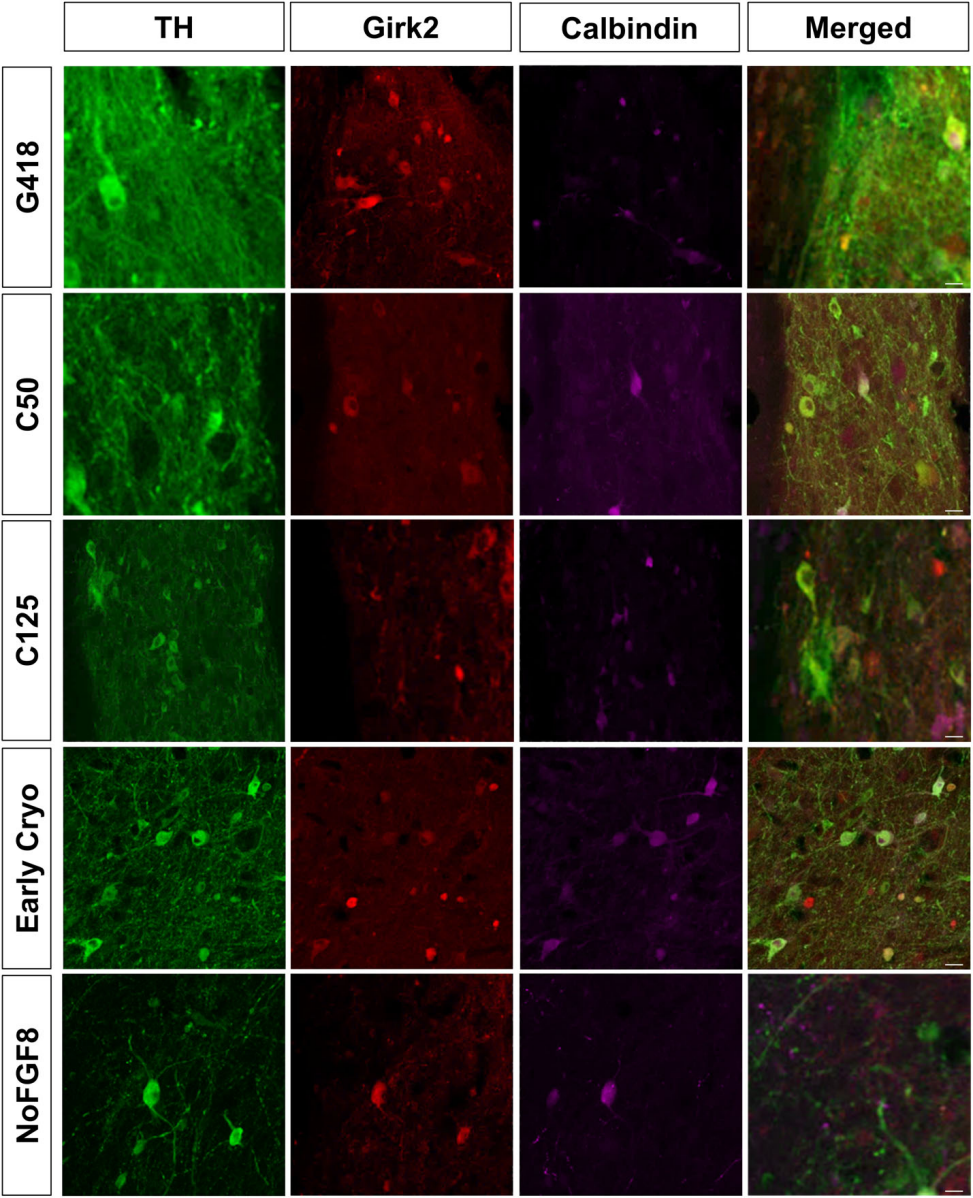
Subtype characterization of midbrain dopamine neurons. Confocal images of representative graft sections triple‐labeled for TH, Girk2, and Calbindin. Confocal microscopic analysis of DA neuronal subtype revealed the presence of A9 subtype DA neurons as indicated by the colocalization of TH/Girk2 cells in all groups with fewer in NoFGF8 and Early Cryo grafts. Some A10 TH/Calbindin cells were present in all groups. While TH/Calbindin‐ir neurons were observed in the grafts, a large number of these also coexpressed Girk2. A small number of the Calbindin‐ir neurons did not stain positive for Girk2, indicating that a majority of grafted neurons across all groups were of the A9 subtype with small amounts of A10 DA neurons. Importantly, NoFGF8 grafts contained clusters of cells which were TH negative, Girk2 negative, but Calbindin‐ir. Scale bar = 25 μm

**FIGURE 6:**
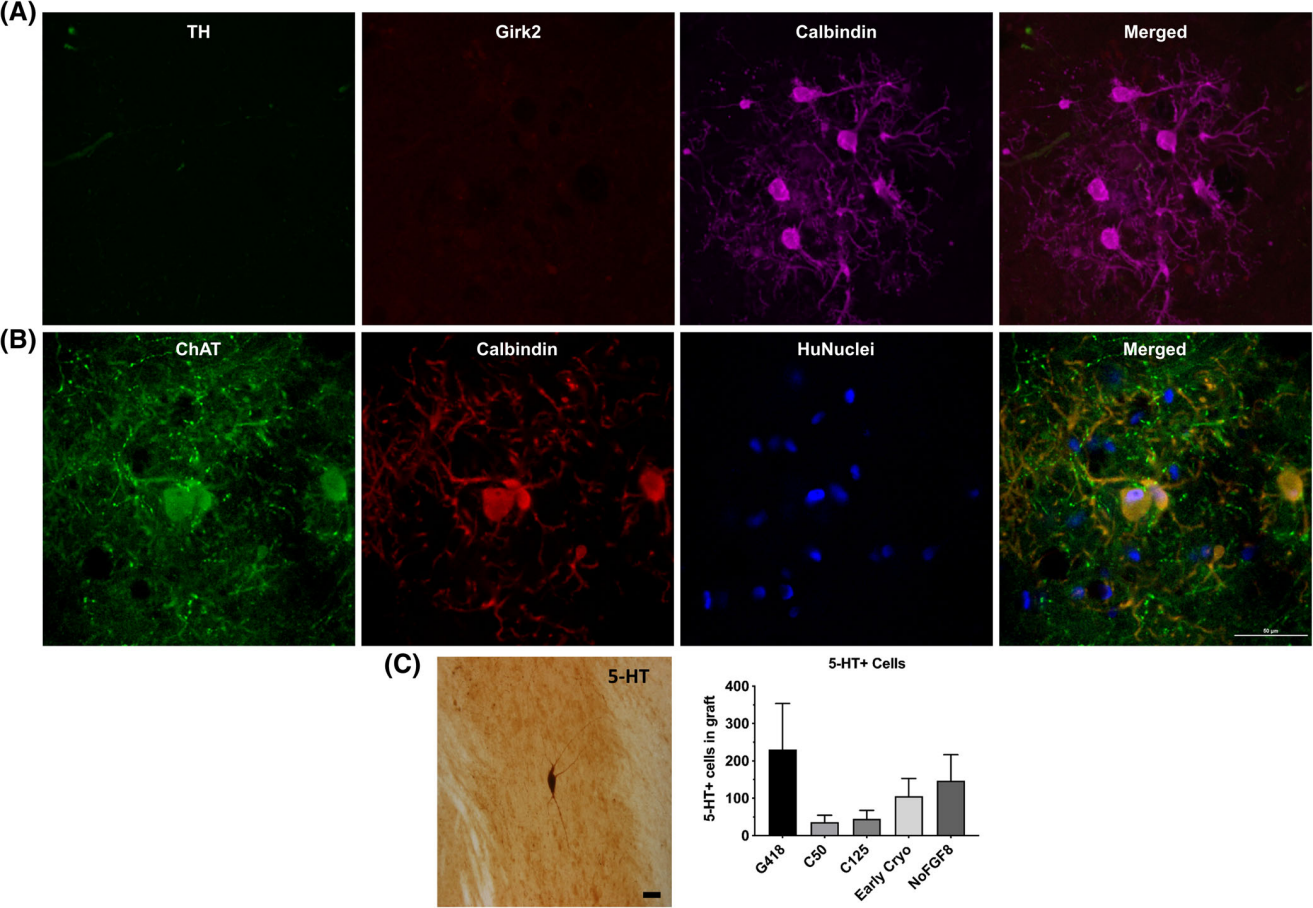
Off‐target cell types. A, NoFGF8 graft sections costained for Th, Girk2, and Calbindin showed clusters of Calbindin‐ir cells that did not express TH or Girk2. B, Triple‐labeling and confocal imaging with ChAT, Calbindin, and HuNuclei revealed that some NoFGF8 neurons coexpressed ChAT/Calbindin/HuNuclei, indicating that these cells differentiated into cholinergic neurons. C, The number of serotonergic (5‐HT) neurons found in grafts was quantified by unbiased stereology. Grafts of G418 contained 230 ± 123; C50 contained 36 ± 18; C125 contained 45 ± 23; Early Cryo contained 106 ± 47; NoFGF8 contained 147 ± 69 5‐HT‐ir cells. No statistical differences were found. Scale bars = 50 μm

To further scrutinize the specific subtype of transplanted DA neurons, we used triple immunofluorescence to distinguish A9 (TH and Girk2 coexpressing) and A10 (TH and Calbindin coexpressing) DA neurons (Figure 5). Qualitatively, across all groups, the majority of transplanted cells coexpressed TH and Girk2, indicating that they had an A9 phenotype. We observed some TH/Calbindin‐ir neurons in the grafts, and a large proportion of these also coexpressed Girk2. Taken together, our observations indicate that a majority of grafted neurons across all groups were of the A9 subtype with small numbers of A10 DA neurons present. Importantly, NoFGF8 grafts contained clusters of cells which were TH‐negative, Girk2‐negative, but Calbindin‐ir (Figure 6A). Further characterization indicated that these neurons were ChAT‐ir/Calbindin‐ir/HuNuclei‐ir (Figure 6B), indicating that some of the grafted cells differentiated into cholinergic neurons. This phenomenon was only seen in NoFGF8 transplants.

Runaway, or graft‐induced, dyskinesias were seen in many patients receiving fetal transplants^30^, ^31^ and it has been speculated that contamination by serotonergic neurons in the graft may contribute to this phenomenon.^32^, ^33^ We assessed whether there were 5‐HT neurons in the grafts, and, using unbiased stereological analysis, found very small numbers (Figure 6C and Table 1) of serotonergic neurons in each transplant (Figure 6C).

## 4 DISCUSSION

Our results show that mDA neurons derived from iPSCs survive long term and completely reverse drug‐induced motor asymmetry when grafted to the striatum of athymic rats with unilateral 6‐OHDA lesions. Furthermore, the data demonstrate that use of MMC as a selection agent during the differentiation process efficiently removes proliferating cells while preserving function of iPSC‐derived mDA neurons after they have been grafted to the brain.

### 4.1 Effects of MMC on cultured neural precursors

The effects of MMC on proliferating cells are well described, with a single DNA cross‐linking event sufficient to kill a bacterial cell.^34^ Using microarray analysis, Felfly et al^35^ previously documented a wide range of changes in gene expression in MMC‐treated (400 ng/mL) neural stem cells. They found 1076 genes with >2‐fold change (up or down) in MMC‐treated neural stem cells. The majority of downregulated genes were related to cell division and metabolic processes, and upregulated genes included those involved in growth factor activity, cell growth, and survival, as well as protein and cellular localization. Of particular interest, MMC‐treated neural stem cells exhibited upregulated mRNA for Map2 and NeuN concurrent with downregulated Nestin mRNA. Furthermore, cell adhesion molecules and nerve growth factor, which are associated with extension of neurites and the mDA neuron phenotype,^36^, ^37^ exhibited increased expression in MMC treated cultures.^35^ Other prior work has focused on the effects of MMC on cultured mDA neurons. Thus, a short pulse of MMC was applied to rodent or primate ESC‐derived dopamine neurons prior to transplantation in order to prevent unwanted proliferation of grafted cells.^38^, ^39^ In our study, the addition of MMC during differentiation eliminated proliferating cells without apparent damage to the neurons, provided that MMC was present at a low concentration over an optimal time frame.

### 4.2 MMC treatment results in functional grafts with minimal proliferation

Importantly, there was minimal cell proliferation in grafts of G418 neurons. Nonetheless, both concentrations of MMC led to even lower numbers of dividing cells. This is consistent with the lack of proliferative cells in the MMC‐treated cells in vitro (Table S1). One caveat is that the Ki‐67 antibody we used was not human‐specific and we cannot rule out that a portion of the Ki‐67‐ir cells were infiltrating glia from the host. Another important observation was the lack of grafted neurons outside the implantation site, indicating that there was little or no migration away from the injection site. Taken together, the data indicating little proliferation or migration in all groups address some potential safety concerns ahead of clinical translation. In addition, although sample sizes were relatively small, there was no evidence that MMC treatment induced transformation or uncontrolled cell growth.

Interestingly, changes in amphetamine‐induced rotations did not appear to be directly related to the number of TH‐ir cells found in the grafts. Grafts of cells harvested “early” in the differentiation process (Early Cryo) had considerably more TH‐ir cells than grafts in the C50 group and yet did not induce statistically significant recovery of motor asymmetry by 8 months post‐transplantation. This disparity demonstrates that while TH expression is necessary, it is not sufficient to guarantee that the neurons have the functional capacity to restore lost dopamine‐dependent functions. In the case of transplanted cells from the NoFGF8 differentiation protocol, there were large numbers of TH‐ir cells (9851‐26 680 per host), but there was little reinnervation of the striatum. It has previously been shown^25^ that axonal outgrowth from grafted neurons is essential for normalization of lesion‐induced behavioral deficits. These results underscore the importance of FGF8 in the development and maturation of dopamine neurons. They also confirm that treatment with a low concentration of MMC is possible without any detectable detrimental effects on the functional capacity of the neurons following subsequent transplantation to the striatum. However, it is important to note that iPSC‐mDA neurons purified using a higher concentration of MMC did not significantly improve behavioral deficits, despite having similar in vitro and long‐term grafting characteristics. The reasons for this are not clear but may suggest that the slightly higher concentration of MMC may have had effects on iPSC‐mDA neuron innervation or functionality that were not detected in the in vitro characterization. Notably, a slightly higher concentration of 500 ng/mL resulted in peeling of the neuron monolayer from culture flasks (Table S1).

### 4.3 FGF8 is necessary for the generation of dopamine neurons that both survive and function after grafting

At the time when we developed our initial protocol for differentiation of iPSCs into mDA neurons, it was argued in the literature that FGF8 was not necessary.^3^, ^4^ Because our process development work leading up to this study indicated that carefully timed inclusion of FGF8 in the differentiation process resulted in improved progenitor expansion, improved cell health, and higher En1 expression, we included FGF8 in the final manufacturing process. We also tested cells manufactured in the absence of FGF8 as a comparator group given that omitting FGF8 from the process has the potential advantage of yielding an almost pure neuron population without requiring a purification step. The differentiation protocol omitting FGF8 generated cultures rich in neurons without proliferation of other cells. The post‐thaw cell health characteristics of these cell preparations were similar to those of cultures where iPSC‐mDA neurons were purified using genetic drug selection. Furthermore, cells grown in the absence of FGF8 demonstrated similar purity, gene expression, and ability to produce dopamine after 2 weeks of in vitro culture. In NoFGF8‐grafted animals, the number of TH‐ir cells in was in line with numbers we found in grafts derived from our revised (including the addition of FGF8) G418 protocol. However, because we found a very large number of surviving HuNuclei‐ir cells in the grafts of the NoFGF8 group, the percentage of TH‐ir neurons was relatively low in the grafts. Moreover, NoFGF8 grafts had the second highest number of 5‐HT‐ir cells in the grafts, and grafts in this group were the only ones containing ChAT‐ir neurons. Taken together, our results show that the NoFGF8 protocol results in suboptimal DA neuron patterning, with higher numbers of other types of neurons surviving in the grafts. These findings are in agreement with published reports, showing that the addition of FGF8 results in improved patterning toward a more caudal mDA neuron fate, reduced numbers of subthalamic nucleus neurons, and improved outcomes after grafting the dopamine neurons to the striatum.^10^, ^28^

## 5 CONCLUDING REMARKS

In summary, we demonstrate that a low concentration of MMC applied during the differentiation process does not impinge on function, survival, or integration of iPSC‐mDA neurons following grafting, while minimizing the number of proliferating cells. The findings also show that inclusion of FGF8 in the differentiation process results in a more robust dopaminergic phenotype. Taken together, we highlight an alternative “drug‐only” method to reduce the number of proliferating cells in grafts of iPSC‐mDA neurons, without the need for genetic modification, which potentially can be employed in clinical translation programs aimed at creating a transplantation therapy for PD.

## CONFLICT OF INTEREST

B.M.H., D.J.M., C.A.T., C.A.C., D.R.W., P.B., C.W.M., and J.H.K. are current or former paid employees or consultants of Fujifilm Cellular Dynamics, Inc. J.H.K. has received commercial support as a consultant from Cellular Dynamics International, Inc, Michael J. Fox Foundation, Abbvie, Exicure, NSGENE, Guidepoint, Inhibikhase, Axovant, and Seelos. P.B. has received commercial support as a consultant from Axial Biotherapeutics, CuraSen, Fujifilm‐Cellular Dynamics International, Idorsia, IOS Press Partners, LifeSci Capital LLC, Lundbeck A/S, and Living Cell Technologies LTD. P.B. has received grants/research support from Lundbeck A/S and Roche and has ownership interests in Acousort AB and Axial Biotherapeutics. R.M.G. declares no conflicts of interest.

## AUTHOR CONTRIBUTIONS

B.M.H., D.J.M.: collection and/or assembly of data, data analysis and interpretation, manuscript writing; C.A.T., R.M.G.: collection and/or assembly of data, data analysis and interpretation; C.A.C.: conception and design, collection and/or assembly of data, data analysis and interpretation; P.B.: manuscript writing; D.R.W.: conception and design, collection and/or assembly of data, manuscript writing; C.W.M.: conception and design, collection and/or assembly of data, data analysis and interpretation, manuscript writing; J.H.K.: conception and design, manuscript writing, final approval of manuscript.

## Supporting information


**Appendix** **S1**: Supporting Information

## Data Availability

The data that support the findings of this study are available from the corresponding author upon reasonable request.
