# Supplementary material for: Mitomycin‐C treatment during differentiation of induced pluripotent stem cell‐derived dopamine neurons reduces proliferation without compromising survival or function in vivo
Source: Stem Cells Transl Med. 2020 Sep 30;10(2):278–90. doi: 10.1002/sctm.20-0014 (PMC7848297; doi:10.1002/sctm.20-0014)
Supplement: Supplementary file 1 — Appendix S1: Supporting Information [file SCT3-10-278-s001.pdf]

Supplemental information

Table S1: Alternative purification methods for iPSC-mDA neurons

Compound	Concentration	Treatment Period (Process Days)	Yield (Fold relative to D5)	Neuron Purity at End of Process (MAP2+/Nestin-)	Neuron Purity Post-thaw (MAP2+/Nestin-)	Notes	Experiment
Amiodarone	1.25 µM	D33-35	3.6	81%	n/d	Low purity; Proliferative cells post-thaw	RND14
Amiodarone	2.5 µM	D33-35	4.3	71%	35% 3dpt	Low purity; Proliferative cells post-thaw	RND14
Ara-C	1 µM	D33-37	2.0	92%	69% 3dpt	Low yield compared to G418; Proliferative cells post-thaw	RND14
Ara-C	2 µM	D33-37	1.6	90%		Low yield compared to G418	RND14
G418	100 µg/mL	D33-38	3.2	97%		G418 control (late treatment)	RND14
G418	100 µg/mL	D26-31	3.6	94% D38	97% D42	G418 control (early treatment)	RND14
None	n/a	n/a	3.9	65%		Large proliferative population	RND14
Ara-C	0.5 µM	D27-31	1.7	93%	88% 3dpt	Proliferative cells post-thaw	RND16
Ara-C	0.5 µM	D34-38	2.1	87%			RND16
MMC	0.5 µg/mL	D34-38	2.2	92%			RND16
MMC	5 µg/mL	D36 (1h pulse)	2.7	90%			RND16
G418	75 µg/mL	D27-31	1.5	93%	94% 3dpt	G418 control	RND16
Ara-C	0.5 µM	D27-31	2.6	81%	76% 3dpt	Proliferative cells post-thaw	RND17
Ara-C	0.5 µM	D33-38	1.2	86%		Low yield	RND17
MMC	10 µg/mL	D29 (2h pulse)	0.8	87%		Low yield	RND17
G418	100 µg/mL	D27-31	1.8	92%	93% 3dpt	G418 control	RND17
None	n/a	n/a	n/d	44%	n/d	Small scale titration; Large proliferative population	RND18
MMC	0.125 µg/mL	D27-31	3.1	97%	n/d	Small scale titration	RND18
MMC	0.25 µg/mL	D27-31	3.1	98%	n/d	Small scale titration	RND18
MMC	0.5 µg/mL	D27-31	2.2	96%	n/d	Small scale titration	RND18
MMC	1 µg/mL	D27-31	0.0	n/a	n/d	Small scale titration; Cell death	RND18
MMC	2 µg/mL	D27-31	0.0	n/a	n/d	Small scale titration; Cell death	RND18
MMC	0.5 µg/mL	D27-31	0.0	n/a		Large scale; Cell death	RND18
MMC	5 µg/mL	D29 (1h pulse)	2.3	96%			RND18
G418	100 µg/mL	D27-31	2.1	98%			RND18
MMC	125 ng/mL	D27-31			83% 3dpt;	No proliferative cells observed	TR1

Compound	Concentration	Treatment Period (Process Days)	Yield (Fold relative to D5)	Neuron Purity at End of Process (MAP2+/Nestin-)	Neuron Purity Post-thaw (MAP2+/Nestin-)	Notes	Experiment
					89% 7dpt; 76% 28dpt	post-thaw	
MMC	250 ng/mL	D27-31			78% 3dpt; 86% 7dpt; 87% 28dpt	No proliferative cells observed post-thaw	TR1
MMC	125 ng/mL	D27-31			88% 3dpt; 82% 7dpt	No proliferative cells observed post-thaw	TR2
MMC	63 ng/mL	D27-31			85% 3dpt; 80% 7dpt	No proliferative cells observed post-thaw	TR2
MMC	50 ng/mL	D27-31			95% 3dpt	No proliferative cells observed post-thaw	DA9LEL
MMC	125 ng/mL	D27-31			84% 3dpt; 87% 7dpt; 88% 28dpt	No proliferative cells observed post-thaw	RND24
NoFGF8	n/a	n/a		77%	85% 3dpt; 90% 7dpt		RND24
n/a = not applicable; n/d = not done; dpt = days post-thaw							

Table S2 Protocol variations. Related Figure 1.

Cell type	Protocol notes
G418	G418 selection to remove non-neuronal cells; cryopreserved on D38
Early Cryo	Same as G418; cryopreserved on D33
C50	50ng/mL mitomycin-C on D27-31; cryopreserved on D33
C125	125ng/mL mitomycin-C on D27-31; cryopreserved on D33
NoFGF8	NoFGF8 expansion phase; no drug selection/purification step; cryopreserved on D26

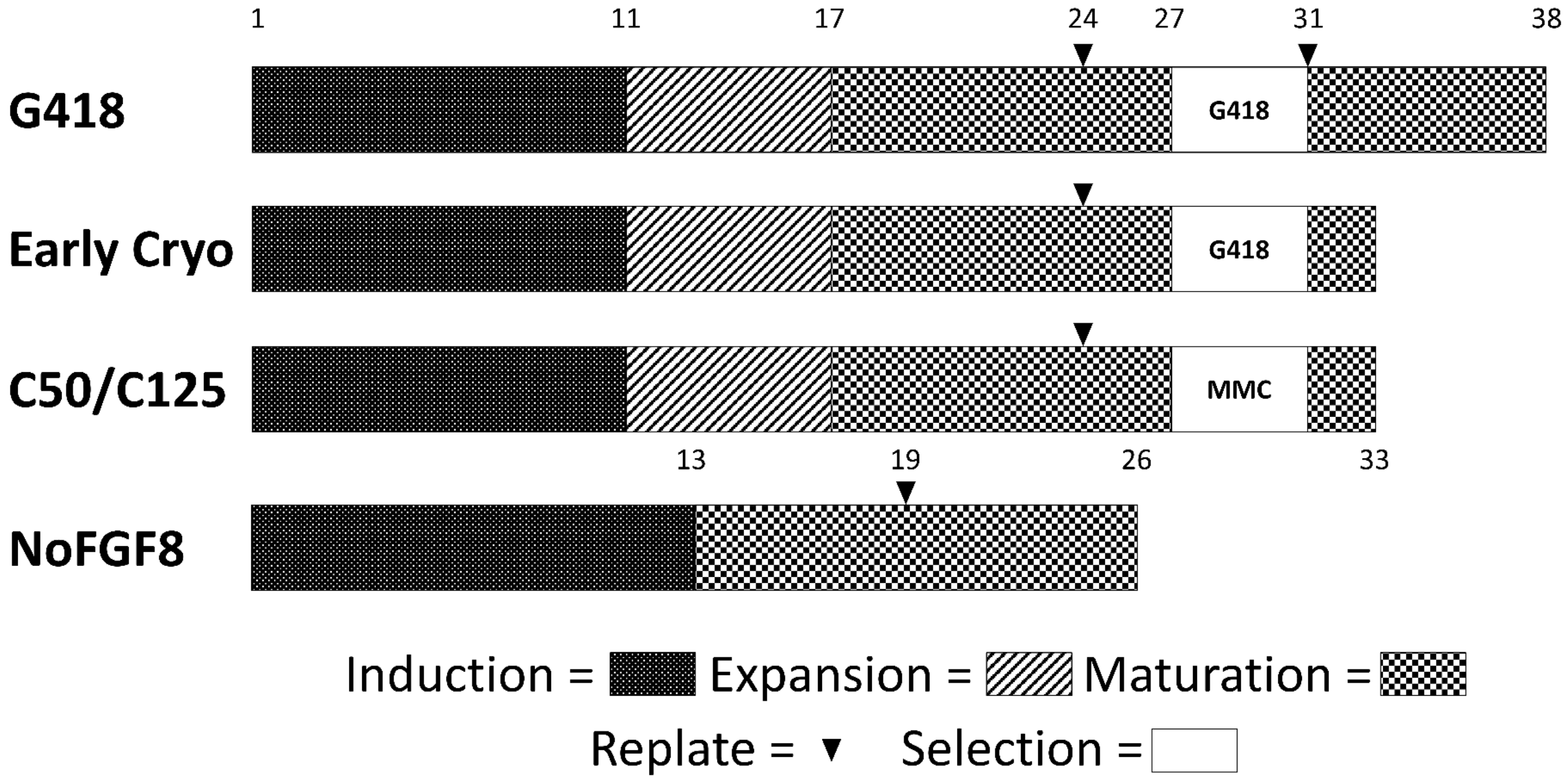

Figure S1 Protocol variations. Related to Figure 1.

Visual depiction of variations of cell preparation protocols. Numbers indicate process day.

Table S3 Antibodies used (IHC, IFC, FACS). Related to Figures 1-6.

Primary Antibody	Species	Company	Catalog #	Dilution	Assay
MAP2	Mouse	Millipore	MAB3418X	1:1000	Flow Cytometry
Nestin	Mouse	BD	560393	1:20	Flow Cytometry
FoxA2	Rabbit	Cell Signaling	8186	1:500	Flow Cytometry
Tyrosine Hydroxylase	Mouse	Sigma	T2928	1:8500 (lot dependent)	Flow Cytometry
Human NCAM (ERIC-1)	Mouse	Santa Cruz	sc-106	1:1,000 (1:500 fluorescent)	IHC
Human Nuclei	Mouse	Millipore	MAB1281	1:800 (1:400 fluorescent)	IHC
Ki-67	Rabbit	Abcam	Ab-15580	1:800	IHC
Tyrosine Hydroxylase	Rabbit	Pelfreez	P40101	1:1,000 (1:500 fluorescent)	IHC
Tyrosine Hydroxylase	Sheep	Pelfreez	P60101	1:500 (fluorescent)	IHC
FoxA2	Goat	R&D Systems	AF2400	1:200	IHC
Girk2	Goat	Abcam	ab65096	1:100	IHC
Calbindin	Rabbit	Cell Signaling	2173	1:300	IHC
5-HT	Rabbit	Immunostar	20080	1:10,000	IHC
ChAT	Goat	Millipore	AB144P	1:100 (fluorescent)	IHC
**Secondary Antibody**	**Species**	**Company**	**Catalog #**	**Dilution**	**Assay**
anti-Mouse	Horse	Vector Labs	BA-2000	1:200	IHC
anti-Mouse	Horse	Vector Labs	BA-2001	1:200	IHC
anti-Rabbit	Goat	Vector Labs	BA-1000	1:200	IHC
anti-Sheep AF-488	Donkey	Invitrogen	A-11015	1:200	IFC
anti-Rabbit AF-488	Donkey	Invitrogen	A-21206	1:200	IFC
anti-Rabbit AF-555	Donkey	Invitrogen	A-31572	1:200	IFC
anti-Mouse AF-647	Donkey	Invitrogen	A-31571	1:200	IFC
anti-Mouse AF-488	Donkey	Invitrogen	A-21202	1:200	IFC
anti-Goat-AF-488	Donkey	Invitrogen	A-11055	1:200	IFC

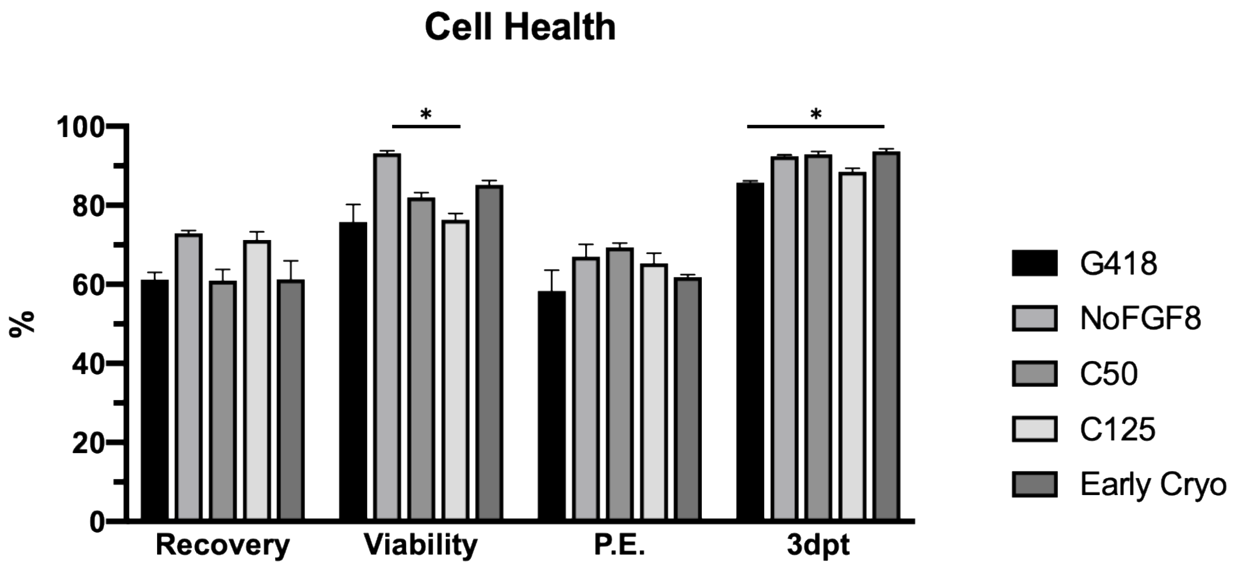

Figure S2 Cell health assessment. Related to Figure 1.

The percent recovery was calculated by dividing total number of viable cells at thaw by the number of viable cells frozen per vial (Recovery). Percent viability (trypan blue exclusion and hemocytometer counting) measured at thaw (Viability) or after plating for 3 days (3dpt viability). The plating efficiency (P.E.) represents the percentage of viable cells that adhere to the surface and remain viable when harvested 3 days later, calculated as the number of viable cells harvested per well at 3 days divided by the number of viable cells initially plated (x 100%). Data graphed as average ± SEM (n = 3 vials thawed in separate experiments). * $p < 0.05$.

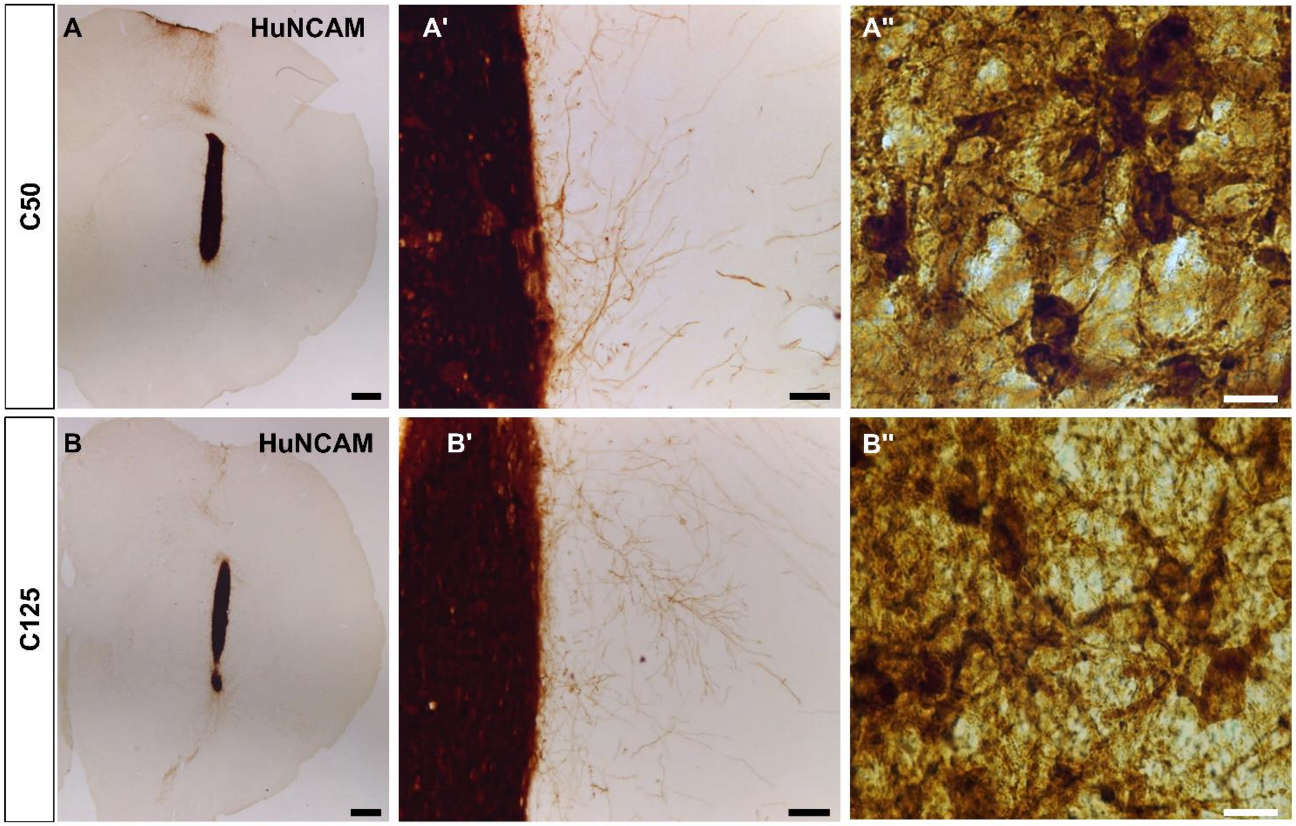

Figure S3 Short term survival in the Cyclosporine-A-immunosuppressed Sprague Dawley rat. Related to Figure 2.

(A, B) Cyclosporine-A-immunosuppressed 6-OHDA lesioned Sprague Dawley rats transplanted with C50 (A) or C125 (B) cells 3 months post-transplantation stained for HuNCAM (A, A', B, B') and TH (A'', B''). Grafts were seen in the dorsolateral striatum with fibers extending from the graft core into the host striatum (A', B'). Scale bar = 500µm (A, B); 50µm (A', B'); 10µm (A'', B''). N = 4/group.

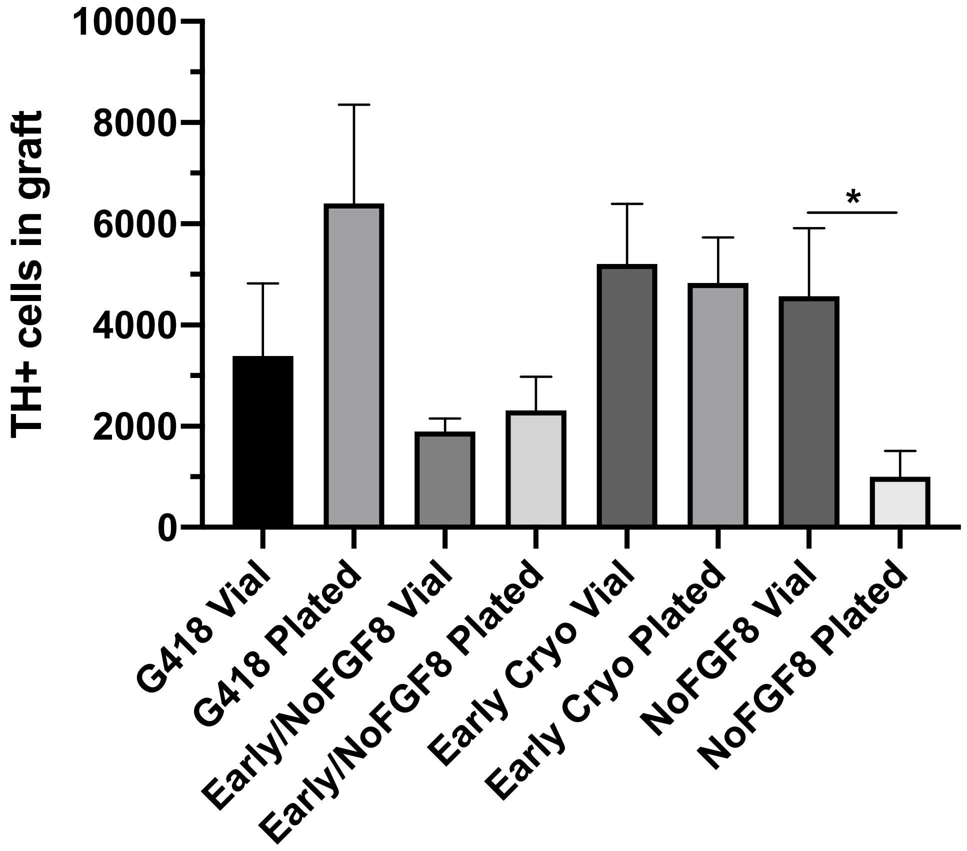

Figure S4 Plating cells 2 DIV prior to transplant does not affect survival. Related to Figure 3.

Intact cyclosporine-A-immunosuppressed Sprague Dawley rats injected with G418, Early/NoFGF8, NoFGF8, or Early Cryo cells either directly from the vial or plated and cultured 2 DIV. Quantification via unbiased stereology of TH-ir cells found 2 weeks after sacrifice revealed the following numbers of TH-ir cells in: G418 3,384 ± 1,435 (vial), 6,401 ± 1,953 (plated); Early/NoFGF8 1,892 ± 266 (vial), 2,313 ± 668 (plated); Early Cryo 5,205 ± 1,191 (vial) 4,829 ± 898 (plated); NoFGF8 4,568 ± 1,341 (vial), 1,007 ± 505 (plated). The only statistical significance found was in NoFGF8 grafts ($p<0.05$). All data are expressed as mean ± SEM with two-tailed T-test. N = 4-8/group.

Supplemental Experimental Procedures

Cell Preparation

Cryopreserved iPSC-mDA neurons were thawed and prepared for transplantation as described previously (Wakeman et al., 2017). Briefly, vials were removed from liquid nitrogen storage and thawed in a 37ºC waterbath, and then diluted and washed according to the manufacturer's protocol (FujiFilm Cellular Dynamics, Inc.). Final resuspension was at 1.5×10^5 in Neural Base Medium 1 with Neural Supplement B (but without growth factor-containing Nervous System Supplement).

For cells cultured 2 days in vitro (2DIV) prior to transplantation in the 2 week survival study, cells were thawed as described and then plated on PLO/Laminin-coated 6-well plates and cultured according to the manufacturer's instructions for iCell DopaNeurons (FujiFilm Cellular Dynamics, Inc.), but with a plating density of 3-4x10^6 viable cells per well. At 2DIV, plated cells were harvested with Accutase (Innovative Cell Technologies, Inc., 30 minutes at 37ºC), washed with the culture medium, passed through a 20µm cell strainer (Millipore Steriflip), and resuspended at 1.5×10^5 in the culture medium.

Flow Cytometry

Cyropreserved iPSC-mDA neurons were thawed, plated on PLO/Laminin-coated 6-well plates, and cultured according to the manufacturer's instructions. At 3 days post-thaw, plated cells were harvested with Accutase (45 minutes at 37ºC), labeled with LIVE-DEAD Fixable Red dead stain (Thermo Fisher), fixed with 4% formaldehyde in DPBS, and permeabilized with DPBS/2% FBS/ 0.1% saponin. Overnight antibody staining and (where applicable) 1 hour secondary antibody staining was performed in the same permeabilization buffer using the antibodies described previously (Wakeman et al., 2017).

Quantitative PCR

Cryopreserved iPSC-mDA neurons were thawed immediately prior to RNA isolation, and real-time quantitative PCR was performed using TaqMan Gene Expression Assays (Applied Biosystems). Procedures and the TaqMan ID numbers for genes assayed have been described previously (Wakeman et al., 2017). Results are expressed as relative expression compared to GAPDH control, with values $<10^{-4}$ considered background.

DA ELISA

Dopamine secretion from cultured iPSC-mDA neurons was measured using a dopamine competitive ELISA kit (Eagle Biosciences) as previously described (Wakeman et al., 2017).

Amphetamine induced motor asymmetry

As previously described (Wakeman et al., 2017), motor asymmetry in rats was assessed by d-amphetamine-induced (2.5 mg/kg, i.p.; Sigma) rotations. After allowing rats to acclimate to the chamber, rotations were tracked for 90-minutes (d- amphetamine), binned every 5-minutes, and average net rotations per minute calculated for each task. A two-way ANOVA (with time after surgery and type of transplant as factors) was performed using Bonferroni's post-hoc test for multiple comparisons.

Only rats that displayed a stable lesion as confirmed by the d-amphetamine-induced rotation task (2.5 mg/kg, i.p.) with a repetitive test-re-test average score of 5 rotations/minute over a 90-minute test period were included for functional analysis. Rats were tested a minimum of three time-points post-lesioning, starting at 3-weeks post-lesioning, to confirm a stable and complete MFB lesion as we have found test-retest variability over time in some animals.

Euthanasia

Rats were anesthetized and perfused transcardially with ice-cold 0.9% saline followed by 4% paraformaldehyde. Brains were removed and post-fixed at 4°C in 4% paraformaldehyde for 18-24 hours before being placed in a sucrose gradient (10%, 20%, 30%) at 4°C and allowed to sink.

Immunohistochemistry

All brains were sectioned into 40µm coronal sections on a frozen sledge microtome and processed for immunohistochemistry using 3,3' - Diaminobenzidine (DAB) with nickel enhancement where applicable or fluorescence immunohistochemistry as previously described (Wakeman et al., 2017). All antibody information can be found in Table S2.

Stereology

A full series of 40μm coronal sections stained for HuNuclei using the DAB method were counted at 60X magnification using Stereo Investigator optical fractionator (Microbrightfield Bioscience, Version10.40). TH (every 12th serial section) and HuNuclei (every 12th serial section) stereological parameters were frame size (75μm x 75μm) and grid size (250μm x 250μm) to count 9% of the total graft area with average CE = 0.13 for HuNuclei (Gundersen m = 1); frame size (80μm x 80μm) and grid size (225μm x 225μm) to count 12.6% of the total graft area with average CE = 0.17 for TH (Gundersen m = 1).

Optical density

Quantification of graft innervation of the host striatum was measured using optical density (Olympus BH2 microscope, Scion Image 1.63). All tissue samples were stained for TH simultaneously to ensure equivalent staining between animals. Every 12th section was stained, the center of the injection site was selected, along with three sections both rostral and caudal (total of 7 sections). The striatum was outlined, excluding the injection site, as the region of interest for measurement. For each hemisphere analyzed, the corpus callosum was measured to obtain background intensity, which was subtracted from each of the corresponding sections. Measurements were also made on the contralateral (intact) hemisphere of all animals.
